# Preventing Religion-Based Hate Crime Victimization Among Youth: A Systematic Review of Personal, Collective, and Policy Responses

**DOI:** 10.1177/15248380241257198

**Published:** 2024-06-13

**Authors:** Sophie Litvak, Janne Kivivuori, Markus Kaakinen

**Affiliations:** 1 University of Helsinki, Finland

**Keywords:** hate crimes, criminology, spirituality and violence, violence exposure

## Abstract

Hate crime victimization targeting the victim’s religious identity poses a serious problem for individuals, communities, and societies. This systematic review describes countermeasures to such victimization, aiming for broad descriptive inclusion by canvassing personal adaptations, collective programs, and institutional-governmental policies. Targeting peer-reviewed articles published between 2002 and 2022, we found 44 articles describing measures related to religion-based victimization prevention. We classified the studied measures into 12 main types. The most salient personal adaptations included camouflage-type blending in to avoid victimization, using religion as a source of resilience, and changing routines to deflect risk. At the collective level, mobilizing community resilience, stereotype reduction, and place-based solutions were often researched. The relatively few institutional-level studies addressed measures to enhance the connection between victims and authorities by various means. The experimental studies heavily concentrated on experiments supporting the efficacy of changing people’s perceptions as a means of prevention. The review concludes with a discussion about research and policy implications.

## Introduction

Due to immigration and mobility globally, many countries are becoming more religiously plural than they used to be (hackett et al., 2015). In turn, this means increasing acquaintance and possible confrontation with individuals and religions that were not previously salient, thus possibly increasing the risk of religiously motivated hate crime victimization (Hackett et al., 2015). In addition to processes of globalization (which can differ in speed from country to country) and increasing religious heterogeneity, the proliferation of conspiracy theories, media portrayals of specific groups, and distinct political events may instigate peaks in religious hate crimes. Examples include the 9/11 attacks leading to anti-Islam hate crimes, and anti-Jewish hate crimes following Israel’s conflicts with Hamas and Hezbollah (Deloughery et al., 2012; Disha et al., 2011; King & Sutton, 2013; Obaidi et al., 2022; Vergani et al., 2022).

In hate crime studies, a hate crime is typically defined as a violent act targeting individuals based on attributes such as language, skin color, religion, societal opinions, or similar characteristics.^1^ This violence is typically (yet, not always) aimed at minorities and can have severe consequences for both individuals and communities (Hardy et al., 2020; Perry, 2001).^2^ When someone is targeted based on their identity, it sends a message to the entire community that they may also face aggression. For this reason, hate crimes are often called message crimes (Chakraborti, 2012). They extend their impact beyond individual victims to entire communities, fostering hostility, violence, and fear (Perry, 2001). Hate crimes take many forms, including intimidation,^3^ threats, property damage, violent attacks, and even homicide (Craig, 2002). This can result in fear and avoidance behaviors, which can create negative cycles when there are no effective guardians in public spaces.

Crime victimization in general has negative mental, physical, and developmental effects on the victims. Although all crimes can be very harmful and distressing to the victim, hate crime has an additional component of targeting the group and the identity of the victim by making it personal and intimate^4^ (Iganski, 2001). Young people who have been victimized in this way are at increased risk for depression and suicidal thoughts (Hardy et al., 2020; Klomek et al., 2008). Moreover, the impact of hate crime victimization on an individual extends to both their physical and emotional well-being, surpassing the effects of parallel crimes that lack the motivating factor of offenders’ hostility toward individuals due to their characteristics (Iganski & Lagou, 2014; Paterson et al., 2019).

Victims can experience feelings of guilt and self-blame (Zempi, 2020). Moreover, individuals who have been subjected to a hate crime are at a greater risk of experiencing sleeping problems and self-confidence issues, panic attacks, anxiety, and depression compared to those who have been victims of non-hate-related crimes. In fact, they are twice as likely to experience these problems following an attack (Home Office, 2009).

In contrast, people are not passive when encountering threats, including hostility toward a religion. Individuals can and do take action to shield themselves from such victimization. They adapt to contexts and places perceived to be dangerous, perhaps trying to hide their religious identity or to project threat to potential offenders (Atwal & Wang, 2018; Flax, 2021; Zempi, 2020). Individuals also draw on their religion to cope (Abu-Ras et al., 2013; Agrawal et al., 2019). Communities can organize to ward off attacks or to become more resilient against such threats (Hargreaves, 2016; Thangaraj, 2021). Often, academic researchers contribute by developing programs intended to reduce risks (Audrey & Batista-Ferrer, 2015; Kayali & Walters, 2021; Paluck et al., 2021).

Similarly, governments can use tools, such as the police and the law, to try to prevent hate crimes or to make it increasingly costly for potential offenders to commit. Thus, the European Union has recently moved “to extend the list of EU crimes to all forms of hate crime and hate speech, whether because of race, religion, gender or sexuality” (European Commission, 2021, p. 1), highlighting the societal and political urgency of preventing hate crime victimization. Prevention methods are also available through initiatives such as the EU-sponsored RAN (Radicalization Awareness Network) collection for radicalization prevention, often within religious contexts (Radicalisation Awareness Network [RAN], 2017). However, these methods tend to focus more on the offender rather than the victim, and they may not address the specifics of religion-targeted hate crimes. Furthermore, such lists and policy thinking typically exclude personal adaptations people take to fend off potential attackers.

In this article, we review research on both informal and formal measures to prevent hate crime victimization. Informal measures refer to any adaptations and reactions potential victims may engage in to prevent possible or further victimization, such as concealing their religion or projecting a threat to potential offenders. Formal measures refer to planned programs and structured victimization prevention interventions developed and funded by organizations, academic researchers, or governmental agencies. Governments and NGOs cannot start endorsing many personal adaptations, such as camouflaging religious identities or seeking revenge, even if these were effective. However, understanding how people or communities defend themselves or adapt to risks can inform the development of programs and policies. Some public policies could *build on* these adaptations, whereas others may aim to *counteract* potentially negative informal reactions such as revenge. Withdrawal and avoidance can also be counterproductive in ensuring safe public spaces. This is something that cannot be known in advance; thus, there is a need to canvass and review the methods and skills people and communities deploy to defend themselves. Just as there are unrecorded crimes unknown to authorities, there may be unobserved preventive measures that could inspire further systematic work toward formal prevention programs.

In this systematic review, we contribute new knowledge on this topic. We first describe our methodological approach and inclusion principles. Then, in the main descriptive section, we report on the adaptations, programs, and policies that have been implemented or found relevant (according to the inclusion criteria) in preventing religion-based hate crimes. Lastly, we tentatively address the question of which measures and adaptations appear promising in terms of efficacy (which will be elaborated further in the text), while focusing on experimental designs. Our perspective and search string involved a focus on young people because prior studies have shown that young people are more prone to experience hate crimes than adults (Kesteren, 2016). Considering the consequences to the victims, this issue deserves attention.

## Prior Reviews

Currently, there is a considerable amount of literature on hate crimes in general, with emerging studies focusing on religion-based hate crime as a research field (Awan & Zempi, 2017; Haynes, Schweppe, & Taylor, 2017; Litvak et al., 2023; Perry, 2001). Farrell and Lockwood’s (2023) review addressed various types of hate crime, emphasizing institutional levels (such as NGOs and other supportive organizations) and legal aspects. Díaz-Faes and Pereda (2022) reviewed the victim–offender overlap in hate crime. These important reviews did not focus on religion specifically. Conversely, the relationship between religion and criminal behavior has been extensively researched, with systematic reviews conducted in this area (Adamczyk et al., 2017; Baier & Wright, 2001). Thus, much less is known about how hate crime victimization is forestalled by people’s spontaneous protective behaviors, and how it could be prevented by specifically planned measures, especially concerning religion-targeting victimization. To our knowledge, there are no prior systematic reviews on this topic. Our contribution to existing research involves exploring individual adaptations, collective actions, and policies to enhance the understanding of hate crime prevention, thereby expanding the current knowledge base in this field.

## Methodology

We use the systematic review methodology (Petticrew & Roberts, 2006) to provide a comprehensive overview of research in hate crime prevention. This review serves as the initial step in surveying the field, encompassing both qualitative and quantitative research. We primarily aim to describe the personal, collective, and policy measures as published research has identified during the last two decades. Due to its descriptive nature, we do not conduct a meta-analysis of effect sizes from primary studies reporting effects (see Adamczyk et al., 2017). By focusing on articles with experimental designs, we highlight effective measures. Our approach adheres to a protocol that was preregistered on the Prospero website.^5^

### Inclusion Criteria

We focused on studies published between 2002 and mid-2022, beginning after the 9/11 terrorist attack. Our search was restricted to peer-reviewed articles in English, excluding so-called gray literature and studies in other languages. The studies needed to address empirically a specific measure against religiously oriented hate crimes, using quantitative, qualitative, or mixed methods. Essays discussing policies without empirical data were not included.

We classified interventions into three main levels. *Personal* interventions are individual adaptive strategies and countermeasures against the risk of religiously motivated hate crimes. *Collective* measures and programs refer to how communities and neighborhoods deploy their collective efficacy to prevent religion-targeting hate crimes. *Institutional* measures refer to the methods governments use, sometimes in connection with NGOs but relying on the public authority. This trichotomy roughly corresponds to personal adaptations, programs, and policies.

In this primary review, we aimed to be as inclusive as possible in describing research on how people navigate the risk of religiously oriented hate crime victimization. Therefore, we included personal adaptations, some of which are illegal in most countries (revenge, counterattack). Regarding more formal interventions, we included measures directly addressing crime victimization and measures believed to target that risk indirectly by manipulating an intermediate mechanism. Prejudice reduction programs are a case in point: If our search string caught the article and presented such a program, it was included in this article even if the strict outcome was not crime victimization risk but rather a mediating mechanism that could lead to hate crimes (such as prejudice, bias, or dehumanization).

Our search string (Supplemental Appendix 1) included studies defining hate differently, such as bias crime, prejudice crime, Islamophobic crime, or antisemitic crime if they targeted religion-based identities. We also included crime against atheists and nonbelievers if explicitly classified as a separate category. Hate speech and microaggression were not used in the search string; the crime part of the string simply specified “crim* OR viol*.” The studies could focus on hate crimes against people with combinations of group characteristics, if one of them was religion (for instance, crimes motivated by gendered Islamophobia). We focused on articles with an emphasis on preventing religion-related hate crime victimization among young people (25 years old or younger). The included articles discussed victimhood, as the search string required, but the interventions or adaptations could also concern other age groups or the entire population. However, all articles not including or not relevant to young people’s victimization (such as articles focusing on the elderly, etc.) were excluded.

### Data Collection

**Figure 1.**
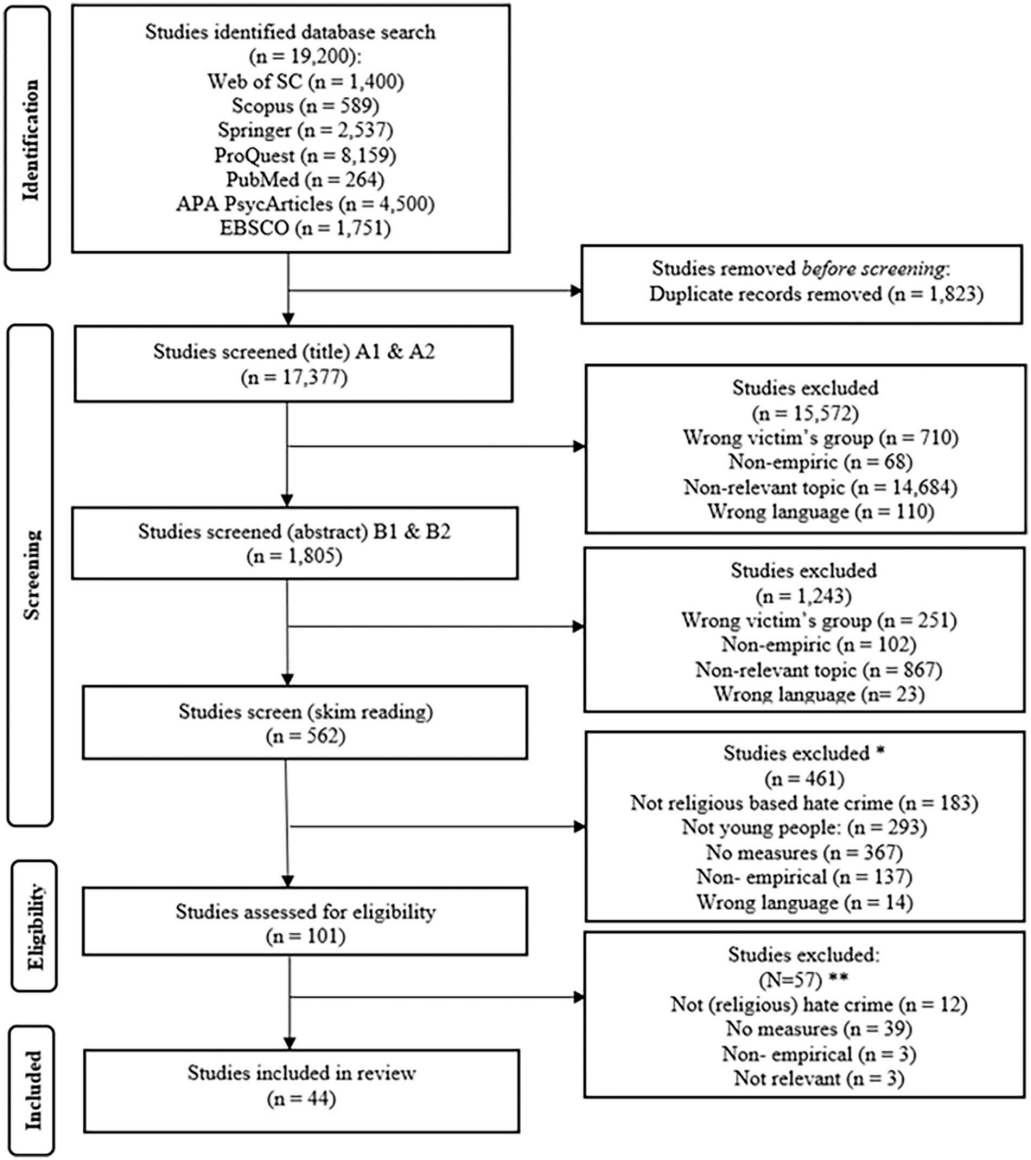
The Preferred Reporting Items for Systematic Review and Meta-Analysis (PRISMA) flow chart of the systematic review (Page et al., 2021). *Will not sum up to the number above, some articles had multiple reasons not to be included **One study included one part of their analysis while the second part was labeled as a standalone article. The first part inspired us to look for the second and include it in the review.

We used seven databases to canvass literature: Web of Science, Scopus, Springer, ProQuest, PubMed, APA PsycArticles, and EBSCO. Before conducting the search, we consulted experts in the fields of religion, victimology, and policymaking in Finland to refine our search terms (see Supplemental Appendix 1). The search took place from June 21, 2022, to July 4, 2022, resulting in 19,200 articles. After a rigorous selection process, 44 articles met all eligibility criteria (refer to Figure 1). Interrater reliability checks were performed during the inclusion and exclusion process to ensure consistency in selecting articles within the intended research domain. Throughout the process, we monitored the reliability of article inclusion through interrater tests and team communication on borderline cases. In the first round of interrater reliability testing (title-based selection, round A1), we achieved 92% agreement on inclusion and exclusion (Cohen’s kappa = 0.73). Following discussions within the research team, the agreement increased to 95% in the next round (A2, kappa = 0.85). During the abstract-level interrater reliability test (round B1), we reached 90% agreement, with a kappa of 0.54. Subsequently, in round B2, we attained 100% agreement (kappa = 1). In the third screening step (eligibility), only the introduction and conclusion of the articles were reviewed, while the full text was examined in the final stage for the eligible articles. The 44 selected articles were coded according to the coding manual. IBM SPSS software (IBM Corp., 2022) was used to describe the final data.

### Aspects of the Research Field

Research on adapting to or preventing religion-based victimization, directly or indirectly via mediating mechanisms, has increased in recent years. Half of the articles were published during this decade and 66% from 2018. This indicates a growing recognition of the issue (whether it indicates increasing religion-based hate crime, cannot be ascertained with our data). Although some articles referenced the 9/11 attacks in 2001, this did not immediately lead to research on religion-linked victimization; perhaps it was initially associated with religion-motivated offending and radicalization. Possibly, research that has traditionally focused on topics such as stereotype reduction may have more recently become intertwined with issues related to victimization and religion.

We questioned what the main research focuses and methods were in the study of measures against religion-based hate crime. Out of the 44 articles, over half (59%) used qualitative methods, mainly interviews and focus groups. Sixteen studies (36.5%) used quantitative research, primarily survey experiments. A few studies (4.5%) used mixed methods. Of the studies, 30% were conducted in the United States, with the United Kingdom following at 1%. If including Canada and Australia, more than half originated from the Anglo-American sphere (59%). Furthermore, 14% of the studies had data from multiple countries. Regarding method–topic alignment, personal adaptations research was mostly qualitative, whereas collective-level interventions research heavily used survey experiments.

Most articles focused on a single religion (64%). However, 27% considered multiple religions, and 4.5% addressed all religions. Some articles did not focus on any specific religion. Islam was mentioned in 73% of articles, Judaism in 30%, Christianity in 20%, and East Asian religions collectively in 25%. Additionally, one article (2%) touched on modern and alternative religions, such as Wicca, Jehovah’s Witnesses, and Scientology. Moreover, atheism was a subject of study in five articles (11%). A single study could cover several religions.

Some articles mentioned multiple preventive measures, leading to their appearance in more than one table and Supplemental Appendix. Overall, the included 44 articles examined 22 types of personal adaptation, 20 types of collective measures, and four types of institutional policy measures (see Supplemental Appendixes 2–4). For each intervention level, we reduced complexity by classifying specific measures as belonging to 12 main techniques of forestalling religion-related hate crime victimization.

## Personal Adaptations

Almost half (47%) of the measures to prevent religion-based hate crime victimization represented efforts taken at the personal level (in 22 articles). We classified these personal adaptations into five major types: blending in, religion as a source of resilience, routine modification, containment, and counterattack (see Table 1 and correspondingly, Supplemental Appendix 2).

**Table 1.** Personal-Level Adaptation Tactics Against Religion-Based Hate Crime Victimization.

Adaptation Tactic	Specific Adaptation Tactic
Blending in (33%)	Religious attire change
	Linguistic adaptation
	Cultural adaptation
	Disguised Affiliations
	Stigma management
Religion as a source of resilience (31%)	Online social in-group bonding
	Protective religious attire
	Social activism
	Collective engagement
	Affiliation Assertion
	Visible Faith Empowerment
	Religious education
	Religious resistance
Routine modification (20%)	Leaving night work
	Spatial alertness
	Faithful Vigilance
	Self-Restrictive Engagement
Containment (11%)	Normalization
	Isolating from religious contact
	Secrecy
Counterattack (5%)	Counterstrike
	Radicalization

### Blending in

One third of the measures (33%) were classified as means of “blending in.” This encompassed various measures, including the removal of visible religious garments such as headpieces, turbans, and shaving beards (Chana, 2020; O’Brien, 2011; Garcia Yeste et al., 2020; Zempi, 2020). Other means of changing the external visibility of religion encompassed linguistic adaptation, where participants adjusted their dialect and language to align with the native population of their current place of residence (Mokoko Gampiot, 2017). Additionally, cultural adaptation was observed, involving adopting local customs, behaviors, and in some instances, practices related to table hygiene (Banerjee et al., 2020). Related tactics included presenting as belonging to a different religion or intentionally concealing a religious identity from others (Cieslik & Phillips, 2021; Morlock, 2021; Wigerfelt & Wigerfelt, 2016). Generally, this tactic to forestall victimization is a form of social mimicry in which a person presents a “front” intended to deflect or deter aggression. Religious mimicry (Smrke, 2004) can take the form of exaggerating religiosity for any tactical purpose or hiding or altering it to deflect unwelcome attention.

Another means of adaptation involves both personal and collective aspects and is known as “stigma management training” (O’Brien, 2011). This article examines the concept of stigma management rehearsal, where religious individuals participate in role-playing within small groups. The purpose is to practice the image they plan to portray to the outside world, mainly to avoid conflict and often to serve as a role model of a model minority. The following section provides a more detailed explanation of these groups, focusing on collective measures. However, this aspect is mentioned here because individuals personally choose how and where to display their role as a model minority and their religion in a manner that contradicts stereotypes and biases while ensuring safety.

### Religion as A Source of Resilience

The second most researched means of personal adaptation related to using religion as a source of active coping and resilience (31%). This amounts to using religion or community connections as preventive and resistance adaptations. Various tactics were observed, including collective engagement and social online in-group bonding where religious individuals established connections with one another to harness collective power and resilience (Agrawal et al., 2019; Flax, 2021; Mercier-Dalphond & Helly, 2021). Social activism was another strategy, involving individuals participating in social initiatives aimed at transforming and enhancing their societal standing as well as reducing vulnerability (Abu-Raiya et al., 2011; Saleem et al., 2022).

Religious resistance emerged as a tactic where individuals drew strength from a robust religious identity, demonstrating pride and resilience when encountering adversity (Mokoko Gampiot, 2017; Hargreaves, 2016). Visible faith empowerment was another approach wherein individuals proudly adorned religious attire to signal resistance and claim their space, thereby normalizing the minority religion (Ben-Moshe & Halafoff, 2014; Perry, 2015). This was a type of mirror tactic to “blending in” (mentioned above). Additionally, the strategy of religious education was implemented, wherein religious individuals sought to educate those unfamiliar with their faith to forestall victimization by showing the person behind the religious label (Bacchus, 2019).

A particularly noteworthy facet within this pattern is the concept of protective religious attire, which one article discussed (Saleem et al., 2022) among other patterns. Despite addressing the social and occupational challenges individuals navigate when wearing religious attire, especially in forms of gendered Islamophobia (in which veiled women are often being treated/targeted differently than Muslim men), the article also included testimony from a veiled woman who emphasized that her religious attire provided protection against public sexualization. She reported experiencing reduced instances of catcalling after she began wearing the hijab. This method of protection also acts as a mirror image of the above-discussed adaptation of hiding religious insignia.

### Routine Modification

One-fifth (20%) of the researched personal measures pertained to modifying one’s routines to avoid religion-based hate victimization. This encompassed various strategies such as avoiding night jobs to mitigate the risk of victimization during commutes (Awan & Zempi, 2020), practicing spatial alertness by remaining vigilant about their surroundings (Chana, 2020; Cieslik & Phillips, 2021; Mercier-Dalphond & Helly, 2021), and engaging in self-imposed limitations on mobility by avoiding certain areas (Garcia Yeste et al., 2020; Zempi, 2020). Moreover, the subcategory faithful vigilance was also categorized under this group, wherein religious individuals exercised spatial alertness while visiting their religious sites (Mokoko Gampiot, 2017; Wigerfelt & Wigerfelt, 2016). Generally, this type of personal adaptation connects to crime risk mechanisms that the criminological routine activity theory describes (Felson & Cohen, 1980). In other words, people engage in situational crime prevention by changing their routines spontaneously, without knowingly applying theoretical assets (Clarke, 1995).

### Containment

We classified three tactics for coping with hate crimes targeting religion as containment: normalization, isolation, and secrecy (11% of the measures). These tactics aim to limit or reduce the incident, sometimes to avoid further victimization. Victims using these tactics seek to modify or manage the situation in a way they believe will contain additional risks or reduce the incidents’ significance. Normalization involves accepting or ignoring hate crime attacks and harassment; those who normalize such events may view them as an unfortunate part of daily life. They may feel powerless to bring about change, question whether retaliatory actions could worsen the situation, or worry that countering hate could reinforce stereotypes depicting religious individuals as deviant or dangerous (Ahmed et al., 2021; Flax, 2021; O’Brien, 2011). Isolation involves victims distancing themselves from the aftermath of hate crimes, and avoiding interactions with both in-group and out-group members (Abu-Raiya et al., 2011). Finally, some bullying victims do not mention the event to their parents as a way to contain it. This decision is based on the belief that reporting the incidents could lead to embarrassment and escalate the situation (Ben-Moshe & Halafoff, 2014). Individuals using this tactic aim to prevent victimization or revictimization by keeping it secret, downplaying it, or normalizing it.

### Counterattack

People use counterattacks as tactics to address the risk of hate crime victimization (5% of personal measures). One such approach is the counterstrike strategy, where individuals respond to actual or potential hate crimes by retaliating, using scare tactics, responding with humor or cleverness, and at times confronting the aggressor physically for deterrence and self-defense (Ahmed et al., 2021; Zempi, 2020). Another example is radicalization, where religious individuals may turn to radical beliefs due to factors such as fear of religious victimization, feelings of inadequacy in protecting loved ones, or a sense of losing control (Ljamai, 2020). However, only one article mentioned this measure.

The articles mentioned above, which explore personal adaptations related to hate crimes, show different levels of directness in their connection to hate crimes. Most of the research focuses on the daily experiences and coping mechanisms of individuals vulnerable to hate crimes based on religion. Other articles shift their attention to experiences of hate crimes, perceptions of hate crimes, and ways to address various forms of school bullying, including bullying related to religion. These tactics are considered potential responses to religious hate crime victimization. The next section will discuss collective measures.

## Collective Measures

By collective measures of counteracting religion-targeted hate crime, we refer to measures the community, neighborhood, school, religious institutions, and other social circles used to prevent religiously motivated hate crime. Perception-changing interventions aiming to reduce tensions between religious communities were also included in this group. We observed five major types of collective measures: mobilizing community resilience, stereotype reduction, place-based prevention, school-based prevention, and increasing positive inter-group contact (see Table 2 and Supplemental Appendix 3). We observed these types of measures in 29 articles. With collective measures, we move toward a heavier emphasis on program types of intervention. These are typically planned and often structured measures to reduce the risk of victimization directly or indirectly. Indirect measures seek to reduce a factor, such as prejudice, which could be a risk factor for victimization.

**Table 2.** Community Programs Against Religion-Based Hate Crime Victimization.

Response Type	Specific Mechanism/Intervention
Mobilizing community resilience (37%)	Safe spaces
	Self-defense training
	Intervention through role-playing
	Community engagement
	Social activism
Stereotype reduction (20%)	Collective praise intervention
	Media literacy intervention
	Positive media exposure
	Meta-humanization and dehumanization intervention
Place-based prevention (20%)	Technological protection of religious spaces
	Spatial protection of religious spaces
	Armed protection of religious spaces
	Criminal background check
	Confidentiality of religious spaces
	Altering the environment
	Armed residents
School-based prevention (13%)	Teachers’ support and involvement
	Interfaith and diversity education
Increasing positive inter-group contact (10%)	Conflict mitigation through sports
	Engagement with interfaith organizations

### Mobilizing Community Resilience

Several articles explored the role of community engagement as a means of resistance against victimization. These studies highlighted the significance of religious or local gatherings, online support, religious associations, the exchange of ideas on victimization prevention, and safe spaces as vital components of this process. The research utilizes qualitative methods, including interviews, focus groups, and participatory observation, to investigate these dynamics. Most of the studies on community resilience related to the Muslim faith (24 out of 29 articles focused on or mentioned this religion).

Saleem et al. (2022) delved into the value of Muslim representation and mentorship, showing how these elements combat feelings of marginalization and cultivate resilience. Participants emphasized their connection to the community as a source of pride and hope, often offering assistance in times of need. Another article (Zempi, 2020) underscored the importance of conversations among veiled Muslim women as a primary source of support, allowing them to share their victimization experiences. Additionally, another study (Agrawal et al., 2019) examined the supportive role of reaching out to family, friends, and neighbors, as well as having safe spaces, community resources, and self-defense training.

A study focusing on Muslim veiled women in Spain (Garcia Yeste et al., 2020) explored the transformation of prejudiced situations into new forms of understanding and trust, offering dialog as a conflict resolution tool. In this context, participants are encouraged to discuss strategies such as engaging in local traditions to bridge divides. This approach recommends an egalitarian nature of dialog, promoting mutual recognition and understanding. Faith-based organizations are seen as potential sources of support and empowerment, enabling participants to challenge racism and anti-Islamic attitudes.

Mercier-Dalphond and Helly’s (2021) research in Canada explored responses to hate crime victimization within the religious community. Various Muslim and women’s organizations provided training and created support platforms. Online spaces and community initiatives were utilized to process the effects of hate crimes and to challenge discriminatory acts. This article emphasized the potential of recording incidents to raise awareness of Muslim victimization. Lastly, another study (Bacchus, 2019) focused on Muslim Americans’ resistance to anti-Islamic attitudes, particularly through community engagement. It suggested that mosques and Islamic centers could serve as supportive institutions, fostering a sense of belonging and promoting coping mechanisms.

The group-based “deep education” that O’Brien (2011) examined aims to educate well-informed stigma managers and members about lessons learned from past or hypothetical hate incidents to illustrate how to address them better in the future. The education groups provide a space for expressing frustration within groups while maintaining outward composure. In other words, besides being a safe space for religious individuals, it is also a place where they can let loose their frustration and anger while remaining in control. Ozalp et al. (2020) assessed, among other objectives, collective efficacy against harmful narratives on Twitter,^6^ indicating that credible actors’ proactive efforts can effectively counter the propagation of negative messages.^7^ On social media, counter-speech from capable sources proves a valuable tool against harmful narratives, decreasing the reach of cyberhate and increasing platform trust.

Overall, these articles suggest that community engagement, religious associations, and safe spaces are used as measures against religion-based victimization risk. Many anti-victimization measures listed in this category share the dimension of social activism. Individuals who draw strength from their religion and religious community engage in online efforts such as counter-speech, online protests, legal campaigns, and sharing accurate religious information to educate and challenge perceptions (Agrawal et al., 2019; Bacchus, 2019; Orjuela, 2020; Saleem et al., 2022).

### Stereotype Reduction

This set of interventions aims to reduce prejudices and negative stereotypes in communities by presenting content with the potential to change people’s attitudes. Murrar and Brauer (2018) explored how entertainment shows could be used to reduce prejudice. In their study conducted in the United States, participants exposed to an educational TV sitcom featuring relatable Arab/Muslim characters (*Little Mosque on the Prairie*) displayed reduced implicit and explicit prejudice compared to those exposed to a control sitcom (*Friends*). This prejudice reduction lasted for up to 4 weeks. A second experiment using a music video further reduced prejudice. Identification with the target group correlated with greater prejudice reduction, suggesting that combining entertainment and education can enhance intergroup relations.

Siem et al. (2021) replicated and expanded the previous study in Germany. They examined mediating effects on prejudice reduction, including perceived group malleability, outgroup variability, and intergroup anxiety. Additionally, they explored how recipients’ levels of right-wing authoritarianism (RWA) might moderate these effects. The video significantly decreased prejudice against Muslims in comparison to control conditions and other interventions. In the United States, identification with American Muslims influenced the intervention’s effectiveness, whereas in Germany, increased perceived malleability and reduced intergroup anxiety mediated the effect. The research shows that, to a degree, that after seeing the video, the prejudice levels among high-RWA individuals were similar to those of low-RWA individuals.

Positive media representation through role models can reduce prejudice, as Alrababa’H et al. (2021) demonstrated in their study on Mohamed Salah, a Muslim soccer player. Salah’s success and public display of his religious identity positively affected perceptions. Hate crimes in England decreased by 16% after he joined the team, anti-Muslim tweets from Liverpool F. C. fans decreased, and an experiment found that knowledge of Salah’s practices increased the belief that Islam aligns with “British values” as perceived by the participants. This suggests that exposure to successful individuals from stigmatized groups can reduce prejudice and thus, potential hate crime victimization.

In her qualitative study based on interviews, Perry (2015) suggested that negative stereotypes associated with the visibility of religious identity (e.g., hijab) can be reversed and used to signify normalcy, especially if media portrayals support this. McCauley (2014) developed strategies to mitigate postconflict bias. Video-based treatments were tested in Côte d’Ivoire with messages from religious or political leaders. The study found that theological messages were effective in reducing bias among Muslims, while messages from political leaders were effective for Christians. These treatments’ effectiveness varied based on recipients’ identities.

Bruneau et al. (2018) studied the effectiveness of the collective blame hypocrisy intervention in reducing collective blame toward Muslims. They compared several videos in an experimental setting to gage attitude changes. The most successful video highlighted the hypocrisy in collective blame, consistently reducing collective blame toward Muslims, anti-Muslim attitudes, behaviors related to vicarious retribution, and potential hate crime victimization. In a similar experimental setup, Moore-Berg et al. (2023) explored the effects of media interventions by comparing various videos aimed at debunking common Muslim stereotypes. These videos addressed media bias against Muslims, shared identity between Muslims (also Americans), emotional distress that media bias triggered, similarities between white and Muslim extremists, the challenges Muslim refugees face, and the benefits of intergroup interaction. The analysis indicated that videos addressing media bias and shared identity were most effective in reducing Islamophobia immediately and 1 month later. This research suggests that media interventions can influence attitudes and potentially impact hate crime victimization.

Gallardo et al. (2022) examined the collective praise method of reducing the dehumanization of a religiously defined group (Muslims). The method involves providing information on the morally praiseworthy acts of religious groups, presenting the stigmatized group first and the nonstigmatized group afterwards. This sequence reduced dehumanization and collective blame against Muslims, supporting the idea that information or media representations can help prevent hostility. The theory behind this effect states that the sequence creates cognitive dissonance, which can influence attitudes. Moritz et al. (2017) investigated changing opinions about Islam through corrective information. Their study suggests that prejudices against Islam may result from knowledge gaps. By providing correct answers and explanations to participants, negative opinions about Islam significantly improved. This study highlights the role of information in challenging and changing prejudiced views.

Pavetich and Stathi’s (2021) study investigated the advantages of metahumanization, which involves perceiving that an opposing group attributes dignified qualities to one’s own group. The study explored both metadehumanization and metahumanization through experiments. It found that metahumanization effectively reduced prejudice, fostered positive attitudes, and indirectly promoted favorable behaviors through humanization. The study presented a model connecting metahumanization with reciprocal outgroup humanization and noted that intergroup threat levels moderated this relationship. The findings suggested that metahumanization can have a positive effect on intergroup attitudes and interactions, even in the presence of a threat.

### Place-Based Approaches

Three studies examined how place- and surveillance-related aspects influence and can be used as protection against, religion-related hate crime victimization.

Based on interviews, Scheitle and Ulmer (2018) interviewed representatives of a wide selection of congregations, exploring the interplay between religious congregations’ intentions of openness and their vulnerability to potential hate crimes. Despite the desire to create an inviting atmosphere, this openness could inadvertently expose the congregation and its members to risks. Addressing their vulnerability might also jeopardize the congregation’s core mission and identity, along with the social fabric they strive to establish and maintain. In response, these congregations take measures to mitigate potential effects on their mission and identity. Some opt to limit attendees’ awareness of the security measures or confine these measures to specific areas, such as outside the worship space, where they pose less threat to the communal reality being cultivated. However, findings highlight a prevailing sentiment that security measures can compromise the sacred ambiance and purpose of a place of worship and thus, can deter people from participating in religious activities.

In the second study regarding religious congregations, Scheitle (2018) surveyed over 1,300 congregations to examine threats and protective measures. Security measures such as alarm systems were common, with Jewish (71%) and Black Protestant (65%) congregations having the highest adoption rates. Security cameras were prevalent, especially in Jewish (54% outside, 39% inside) and Muslim (66% outside, 53% inside) congregations. Jewish (72%) and Muslim (66 %) congregations were significantly more likely to adopt multiple security steps. Jewish and Muslim congregations confront heightened hate-motivated risks, experiencing more threats with perceived bias motivation. Their greater fear of victimization, particularly violent forms, prompts them to implement heightened security measures. The studies mentioned described the place protection needs and activities among religious denominations, but they did not evaluate their effectiveness.

Dhattiwala (2016) examined the conflict between Hindus and Muslims by using a unique research design, comparing neighborhoods within the same electoral ward in Ahmedabad, and highlighting the role of ecology in microspatial violence patterns. The research integrates the cost–benefit analyses to explain attackers’ choices. The research indicates that two crucial ecological factors—the built environment and the distribution of potential targets—heavily influenced attackers’ decisions and outcomes. Vulnerability was higher where escape was impeded. For Muslims, vulnerability was greatest when they were concentrated in small numbers and had obstacle-free routes for attackers. Where escape routes existed to larger Muslim concentrations, looting and arson occurred instead of killings. The built environment, itself historically connected to socioeconomic structures, influences how motivated offenders and suitable targets interact in urban spaces. Attackers demonstrated strategic target selection influenced by risk aversion. The built environment shaped both attackers’ and targets’ behavior. Narrow lanes increased risk aversion for vehicle-based attackers, affecting their strategy. Muslim residents exploited their spatial settings for emotional dominance during attacks, altering the dynamics. In essence, this study reveals that even in extreme situations, spatial factors and ecological dynamics play significant roles in influencing violent actions and outcomes within communal conflicts. Thus, in principle, crime prevention through environmental design might work to reduce religion-based hate victimization.

### School-Based Prevention

Several articles explored teachers’ role in preventing hate crimes, religious education, and addressing religiously motivated bullying in schools. Based on interviews, Ben-Moshe and Halafoff’s (2014) research emphasized the urgency of educational interventions to counter antisemitism and promote religious understanding in Australian schools. They highlighted the need for teacher education on cultural diversity and Judaism, and they advocated for collaboration between schools and the Jewish community. Berger et al. (2015) tested the ERASE-Stress-Pro-Social (ESPS) program’s impact on Jewish Israeli students who experienced trauma during the 2008 Gaza War. The study found that the ESPS intervention significantly reduced trauma-related distress and improved attitudes toward diversity between different religious groups. Dhali et al. (2022) examined the Ethics and Religious Culture (ERC) program’s effectiveness in promoting students’ ethical development and resilience against extremism. The study indicates the ERC program enhances open-mindedness toward other viewpoints and religions as well as critical thinking. Savage et al. (2014) introduced the Being Kenyan Being Muslim program, which aimed to promote value complexity and to counter extremism in Kenya. The study demonstrated the program’s success in increasing integrative complexity and fostering appreciation for diverse viewpoints. Finally, Savani et al. (2020) focused on social work education and underrepresentation of Muslims. Their intervention positively affected student knowledge and attitudes about Islam highlighting the need for reliable instruments in this area.

### Increasing Positive Contact Between Groups

Several studies have examined the role of increased contact between religious groups. Galily et al. (2013) studied sports initiatives in Israel that aimed to improve Arab–Jewish relations. Programs, such as the Mifalot Program, Peres Center for Peace, and Friendship Games, promote positive interactions between youth from different backgrounds through sports. The research showed that participation in these initiatives reduced hatred and increased trust among participants. In a study on interfaith dialog, Agrawal et al. (2019) examined coping mechanisms of Muslims in the United States. The study advocated for active engagement with the broader community to break down isolative groups, promoting collaboration and harmony across cultures. Similarly, Shankar and Gerstein (2007) explored peacebuilding solutions for the Hindu–Muslim conflict. Participants suggested strategies, such as community gatherings and joint celebrations to build trust and collaboration, with a shared aspiration for peaceful coexistence and a better future. Similarly, Orjuela (2020) addressed anti-Muslim sentiments Buddhist monks perpetuated in Southeast Asia. The article highlighted interfaith dialog as an approach to counter hate speech and violence. In this approach, religious leaders and activists engage in conversations and activities that promote understanding between groups. While some challenges exist, these initiatives contribute to attitude changes and improved relationships among participants, challenging biases and promoting coexistence.

## Institutional-Governmental Policies

By institutional measures, we mean programs initiated by government authorities using legal-administrative interventions such as the police force, legislation, and government policy programs. This category also includes community actions that the state established or funded, often involving multiprofessional cooperation. We identified three articles with four measures, detailed in Supplemental Appendix 4. Our inclusion criteria required empirical data, excluding articles that solely discussed regulations or sanctions from a legal perspective. Although such articles were identified in our search, they were more general in scope compared to those focusing on personal adaptations and collective responses. Regarding content, these measures sought to enhance penal certainty through police training and by improving the relationship between victims and authorities.

### Police Training

Two articles were identified regarding police competencies and training for addressing hate crimes. Hardy et al. (2020) used questionnaires, in-depth semistructured interviews, and observations to examine hate crime-related training in the police force. The study lacked an outcome variable related to hate crimes in general or religion-related hate crimes specifically. However, their data indicate that trainees are more likely to understand and apply their learning to their work when they experience interactive, engaging, and creative training methods that encourage debates and discussions. In an article with a similar process evaluation approach, Jurczak (2019) evaluated Polish police officers’ skills, knowledge, and qualifications related to hate crimes by qualitatively analyzing accessible hate crime training programs. The research revealed that while officers may not have absorbed all available information from the hate crime training, the training had a positive effect on their attitudes and awareness of hate crimes. Both studies focused on hate crimes in general, with a mention of religion as one triggering factor for such crimes.

### Victim Reporting and Acknowledgment

Chana (2020) conducted qualitative interviews to evaluate three policy efforts in the United Kingdom: third-party reporting centers allowing victims to report crimes to an entity not associated with the police, local intensification of control through community–police cooperation and relations, and recognizing and detecting displaced victimization. The latter pertains to the issue of unnoticed victims resulting from displaced targeting, such as Sikhs or Hindus who are targeted by offenders assuming they are Muslims. Enhancing the certainty of formal control could be achieved by considering such mistargeted victimization in the provision of protection and services. In summary, the study emphasizes the potential and challenges in public–private collaboration for crime prevention, including in the realm of religion-based hate crimes. It also highlights the issue of underreporting, which undermines the effectiveness of deterrence in preventing religion-based hate crimes.

## Observations on Efficacy

This article primarily aims to describe broadly the ways to prevent religion-based crimes, whether directly or indirectly, through key mechanisms. Simply describing a method or tactic does not demonstrate its effectiveness. To assess the effectiveness of measures, causal inference is needed, with the gold standard being experimental design. Therefore, we tentatively explored the effect by examining studies that used an experimental design. Of the studies included, 13 (30%) utilized some form of randomized controlled trial. Except for positive religious coping as a personal adaptation, all studies evaluated structured programs at a collective level. The studies were conducted in six countries (the United States, the United Kingdom, Germany, Israel, Cote d’Ivoire, and Kenya) and mostly relied on survey experiments. None of the studies considered crime victimization as a direct outcome variable; instead, they focused on indirect mechanisms, primarily prejudice reduction. Interestingly, all experimental studies showed positive effects of the interventions examined, with one reporting mixed results. Mobilizing community resilience, stereotype reduction, school-based prevention, and enhancing positive intergroup contact were among the main types of collective measures that had received support from experimental designs. These programs typically aimed to change how individuals perceived other groups by helping them unlearn negative perceptions or adopt positive ones. The stimuli often involved media content or intergroup contact. However, there were no evaluations of the effects of personal adaptations and policy interventions.

## Discussion

In this systematic review, our main aim was to identify the various activities and measures that have been studied to prevent religion-based hate crimes. The review includes informal measures and formal responses, such as personal adaptations, collective-level measures, and institutional/governmental policies. By exploring a wide range of approaches, we aimed to identify effective strategies for prevention. For example, official prevention programs could benefit from studying personal adaptations to develop more efficient policies. However, illegal actions, such as revenge or counterattacks, cannot be recommended for prevention. Similarly, personal responses such as avoiding risky places or changing language use cannot be endorsed in public policy due to ethical considerations. Public authorities strive to ensure equal rights and access to public spaces for all religious groups. Informal responses such as religious coping or religion as a source of resilience could potentially inform official policies.

### Descriptive Results

We found 44 articles studying 46 measures empirically to prevent religion-based hate crimes. To simplify, we categorized these measures into 12 main types for preventing victimization due to religion-related hate crimes.

Five of these main types involved personal adaptations. “Blending in” referred to individuals attempting to conceal their religious identity to avoid victimization, using social mimicry as a defense strategy. “Religious resilience” involved efforts to transform religious identity into a source of strength. “Routine modification” described behaviors such as avoiding unsafe areas to protect oneself from victimization. “Containment” is associated with acknowledging and accepting the threat, whereas “isolation” refers to isolating oneself from both the in-group and the out-group. In addition to these more passive adaptive strategies to religious threats, the literature also discussed more active approaches such as counterattack and revenge. Among the studies on personal adaptations, all except one were qualitative. The exception was the study on religious coping with hate crime threats (Abu-Raiya et al., 2011), which evaluated the effectiveness in terms of mental health outcomes. This study indicated that resilience through positive engagement with fellow believers could help mitigate the effects of hate crime victimization and serve as a coping mechanism against potential future attacks. The second main category, collective responses, included both spontaneous mobilization of community resilience and developed programs, some of which were tested in randomized controlled trials. Descriptively, we classified these measures into five categories. Mobilizing community resilience referred to measures such as community engagement and social activism, which also included self-defense training and role-playing. Stereotype reduction included several methods to combat prejudice in communities. Place-based prevention drew on the well-known mechanisms of situational crime prevention. Other measures included school-based prevention and increasing positive intergroup contact. Taken together, collective responses against victimization threat connect to theories of collective efficacy: trying to mobilize the resilience inherent in communities.

The third level of interventions, the institutional-governmental level, was, perhaps surprisingly, the least researched in our review (the methodological reasons for this will be described below). We observed three studies that linked penal certitude. The dimension of penal certitude refers here to improving the connections between the authorities and the victims by means such as police training or easing victims’ way to report incidents.

We also tentatively explored the effects of different measures by examining experimental studies. These studies mainly focused on collective-level structured programs that targeted indirect mechanisms such as stereotype reduction. One effective measure identified was positive religious coping, which showed positive effects on mental health outcomes and resilience. Other responses studied included perception change through media, role models, familiarity, projection of the same values to the in-group and the out-group, and actual physical contact, all showing promising results in combating prejudice and potential hatred. Furthermore, stereotype reduction (Table 2) such as collective praise, media literacy, positive media exposure, and metahumanization, combined with school-based prevention might show promising results. Previous studies and reviews on antisocial behavior and radicalized thinking indicate that the formation of antisocial tendencies begins at an early age, and they argued for intervention at those ages (Beelmann & Heinemann, 2014; Beelmann & Lösel, 2021; Raabe & Beelmann, 2011). Therefore, implementing interventions at a school age, where scholars do not need to search for individual participants but rather contact schools for permission and inquire if they could influence young and possibly biased minds, could be effective. Additional studies showed that communication, appropriate training, and education regarding diversity conducted with the police could be highly promising. Although the studies mostly criticized the outcomes and yielded mixed results, they showed that the understanding of the importance of hate crime victimization, the understanding of the victims’ perspectives, and tolerance improved among police officers after various hate crime training programs. The most critical findings can be seen in Table 3.

**Table 3.** Critical Findings.

**Critical Findings**
Research output relevant to preventing religion-based hate crime victimization has increased over the recent years.
Most research has been conducted in the Anglo-American sphere, focusing on Islam and Judaism as targets of hate.
Five main tactics of personal adaptation to risk include blending in, religion as a source of resilience, routine modification, containment, and counterattack.
At the collective level, the main measures include community resilience, stereotype reduction, place-based prevention, school-based prevention, and positive intergroup contact.
At the level of governmental policies, links between victims and authorities have been central topics.
Effects studies have mostly assessed measures to reduce negative beliefs such as prejudice, supporting their efficacy as indirect means of reducing religion-based hate victimization.

## Limitations and Research Needs

We excluded research published in languages other than English, which may have left out specific victim groups or situations where hate crime victimization occurs. Gray literature was also excluded. This may partially explain why we found few studies on governmental policies. Our focus was on victimization, and future studies could explicitly address the role of the offender. Additionally, while our primary emphasis was on examining hate crime victimization among young individuals, several studies included youth along with other groups. An illustration of the implications of the review for practice, policy, and research can be found in Table 4. The study of personal adaptations was primarily qualitative, and it did not focus on effects and outcomes. This does not reduce the adaptations’ significance but emphasizes how individuals develop strategies to address victimization, recognizing that these approaches’ effectiveness is subjective. Understanding how people naturally respond is valuable for prevention efforts. For instance, misidentifying someone’s religious affiliation (Chana, 2020) can lead to exclusion from support systems. Therefore, insights into personal adaptations can inform public policies. Another example might be telephone services that support the “fake phone call” tactics of women creating the impression that they are not alone while walking in public space at night. Thus, qualitative studies are highly useful for developing public and collective-level responses.

**Table 4.** Implications of the Review for Practice, Policy, and Research.

**Implications of the review for practice, policy, and research**
To prevent religion-based hate crime victimization, a stronger connection between policymakers and academia is needed.
Future research should be more inclusive in terms of religious groups, and it should include nonbelievers as targets of hate.
More research is needed from countries other than the United States and the United Kingdom.
While some informal tactics of adapting to risk cannot be recommended in public policy, it is important for policy developers to know how people spontaneously protect themselves from victimization to build on such adaptations or to forestall counterproductive adaptations.
Stereotype reduction and other cognitive interventions appear as promising means of preventing religion-targeting hate crime victimization.
Future studies should use outcomes related more directly to criminal behavior or victimization, in addition to capturing indirect mechanisms such as biased cognition.
More research is needed on how governments can prevent religion-based hate crime victimization by means of criminal justice and enforcement; quasi-experimental methods might be used to study the effects.

The field of studies on adaptations and measures against religion-related victimization is primarily focused on Islam, with Judaism ranking second. This likely reflects the significant immigration from North Africa and the Middle East to the West, Middle Eastern conflicts that can escalate hate incidents in Europe and the United States, and jihadist terrorist attacks in the West that may incite anti-Islam hate crimes. While these focuses are justified, further studies could explore how to prevent victimization among nontraditional religions, nonbelievers and other religiously diverse groups. Understanding what strategies are effective for different groups may vary depending on the context. Even studies involving small religious groups could provide insights for prevention (consider the case of the Amish, who primarily seek to practice their religion freely without interference; Byers, 2008). The research methodology can also have an effect on the results concerning religious groups (as well as other features of hate crimes). For this reason, we have wanted to be as explicit as possible regarding our methodological choices.

The current study focuses on the period preceding the Hamas attack on October 7th, 2023, and the following conflict. As these events increased antisemitism around the world (Hare, 2023; Robinson-divine, 2024), more research is needed on antisemitism, and the resolution of the conflict between Judaism and other religions.

Regarding crime prevention in the field of religion-based hate victimization, one important finding is the lack of studies on institutional-governmental policy levels. One reason for this may be the focus on articles with clear empirical data sets. Much of the literature on formal governmental policies, such as legislative changes, tends to be essayistic, mainly describing introduced changes and speculating on their effects. Another factor is the limitation to peer-reviewed articles; future studies should consider including gray literature from administrative sources. Additionally, as the articles on that level showed, constraints such as limited operational hours, training resources, and deficient communication channels between law enforcement and affected communities may contribute to the diminishment of attention and resolution dedicated to preventing hate crime victimization. Finally, our search criteria required the mention of the victim, which excluded purely offender-focused governmental approaches.

Regarding policy evaluation, experimental studies mostly involved structured programs designed for collective-level interventions. This is a strong research program that often deploys survey experiments studying concepts such as prejudice. Studying actual criminal behavior with robust designs may be challenging; such behavioral studies will be needed in the future. Thus, we cannot conclusively say that collective-level interventions on perceptions are effective while personal and institutional interventions are not. Rather, we lack evidence on the effects of the latter type of measures. This critique might have broader implications because it is challenging to conduct experimental designs to study how social-structural or political factors or reforms might reduce conflict and religion-based victimization. There may be a need to expand the methodological approaches to include quasi-experimental designs and natural experiments.

Notwithstanding these limitations, beneficial and notable patterns emerge across all three levels of forestalling religion-related hate crime. The studies we reviewed yield insights for future research and policy development. While acknowledging the absence of a one-size-fits-all solution (especially while looking at hatred toward different religious groups, who possess different histories, ideologies, demographics, associated ethnicities, and roots) certain recurrent patterns, particularly from the intervention perspective, can be explored and enhanced for further implementation. Including the personal adaptations of people implies that the victims’ everyday struggles and solutions are acknowledged and they are given a voice in the search for solutions (Chakraborti & Hardy, 2017).

## Supplemental Material

sj-docx-1-tva-10.1177_15248380241257198 – Supplemental material for Preventing Religion-Based Hate Crime Victimization Among Youth: A Systematic Review of Personal, Collective, and Policy Responses Supplemental material, sj-docx-1-tva-10.1177_15248380241257198 for Preventing Religion-Based Hate Crime Victimization Among Youth: A Systematic Review of Personal, Collective, and Policy Responses by Sophie Litvak, Janne Kivivuori and Markus Kaakinen in Trauma, Violence, & Abuse

## Notes

1. In this article, we build on the definition of hate crime commonly used in this research field, rather than on legal definition. What is protected by law might vary across countries and might include/exclude disability, class, caste, subculture, and poverty in addition to gender, race, religion, ethnicity, and sexual orientation.

2. In numerous legal frameworks, the classification of a crime as a hate crime does not inherently require a premeditated intent to inflict harm based on a protected characteristic. Rather, the criterion for such categorization often hinges upon the manifestation of hostility toward these protected characteristics during the execution of the offense.

3. However, it is crucial to emphasize that not all forms of critique, questioning, negative commentary, or deviant opinions regarding a religion automatically constitute a hate crime.

4. Nevertheless, a recent study showed that hate crime victimization hurts some individuals more than others, and it cannot be completely generalized (Mellgren et al., 2021).

5. See https://www.crd.york.ac.uk/prospero/display_record.php?RecordID=330235.

6. Now known as X.

7. We included this article in this review because the authors also looked at the online–offline spread of the negative behaviors.
