# Supplementary material for: Preventing Religion-Based Hate Crime Victimization Among Youth: A Systematic Review of Personal, Collective, and Policy Responses
Source: Trauma Violence Abuse. 2024 Jun 13;25(5):3696–712. doi: 10.1177/15248380241257198 (PMC11545204; doi:10.1177/15248380241257198)
Supplement: sj-docx-1-tva-10.1177_15248380241257198 – Supplemental material for Preventing Religion-Based Hate Crime Victimization Among Youth: A Systematic Review of Personal, Collective, and Policy Responses [file sj-docx-1-tva-10.1177_15248380241257198.docx]

# Appendix 1

The search string used in the systematic review of prevention of hate crime victimisation

| *Required topical dimensions:* | | | | | | | | |
| --- | --- | --- | --- | --- | --- | --- | --- | --- |
| *Religious groups* |  | *Hate motive* |  | *Crime* |  | *Victim focus* |  | *Policies and adaptations* |
| *relig* OR *non-relig* OR | **AND** | hate* OR | **AND** | crim* OR | **AND** | victim* OR | **AND** | prev* OR strateg* OR reduc* OR adapt* |
| *nonrelig* OR *anti-relig* OR |  | bias* OR |  | violen* |  | against* OR |  | OR avoid* OR cope OR coping* OR self* |
| *antirelig* OR *islam* OR |  | prej* |  |  |  | target* OR |  | OR program* OR Interv* OR training* OR |
| *muslim* OR *anti-semi* OR |  |  |  |  |  | surviv* OR |  | campaign* OR approach* OR collec* OR |
| *jew* OR *juda* OR *budd* OR |  |  |  |  |  | fatal* OR |  | *commun* OR kehillah* OR initia* OR |
| *hind* OR *christ* OR *cathol* |  |  |  |  |  | injur* |  | polic* OR penal* OR deter* OR sanction* |
| OR *protest* OR *orthod* OR tao* |  |  |  |  |  |  |  | OR guard* OR cctv OR treat* OR reform* |
| OR dao* OR *sikh* OR *confuc* |  |  |  |  |  |  |  | OR school* OR responder OR *work* OR |
| OR *shint* OR *baha* OR anti* OR |  |  |  |  |  |  |  | service OR mediat* OR restorat* OR |
| *athei* OR *agno* OR *mormon* |  |  |  |  |  |  |  | therap* OR councel* OR mentor* OR |
| OR *satan* OR *pagan* OR |  |  |  |  |  |  |  | reveng* OR retaliat* OR vigilant* OR |
| *wicca* |  |  |  |  |  |  |  | neighbo* OR turf* OR defen* |

# Appendix 2

Descriptives of studies on personal adaptations

|  | **Author and publication date** | **Country (data)** | **Method** | **Religion (preventing victimisation against:)** | | | | | | | **Main adaptation / intervention type**** | **Number of measures frequency** | | **Specification of main outcome** | **RCT** | **Result if RCT** |
| --- | --- | --- | --- | --- | --- | --- | --- | --- | --- | --- | --- | --- | --- | --- | --- | --- |
|  |  |  |  | *Christ.* | *Islam* | *Judais* | *Asian* | *Other* | *Nonrel.* |  |  |  |  |  |  |  |
| 1 | Abu-Raiya et al. (2011) | USA | Mixed |  | 1 | | |  |  | Religion as source of resilience (2), containment (1) | | | 3 | Response to and experience of hate crimes victimisation | No | .. |
| 2 | Agrawal et al. (2019) | USA | Qualitative |  | 1 | | |  |  | Routine modification (1), religion as source of resilience (2) | | | 3 | Response to and experience of hate  crimes victimisation | No | .. |
| 3 | Ahmed et al. (2021) | USA | Qualitative |  | 1 | | |  |  | Blending in (1), containment (1),  counterattack (1) | |  | 3 | Response to and experience of hate  crimes victimisation | No | .. |
| 4 | Awan & Zempi (2017) | UK | Qualitative |  | 1 | | |  |  | Blending in (1) | |  | 1 | Perceived safety  from hate crime victimisation | No | .. |
| 5 | Awan & Zempi (2020) | UK | Qualitative | 1 |  |  | 2* |  | 1 | Routine modification (1) , blending in (1) | | | 2 | Response to and experience of hate  crimes victimisation | No | .. |
| 6 | Bacchus (2019) | USA | Qualitative |  | 1 | | |  |  | Religion as source of resilience (1) | | | 1 | Response to and  experience of hate crimes victimisation | No | .. |
| 7 | Banerjee et al.  (2020) | Multinational | Qualitative |  | 1 | | |  |  | Blending in (3), religion as source of  resilience (1) | | | 4 | Reduction of biased  bulling | No | .. |
| 8 | Ben-Moshe & Halafoff (2014) | Australia | Qualitative |  |  | 1 | |  |  | Containment (1), religion as a source of resilience (2) | | | 3 | Response to and  experience of hate crimes victimisation | No | .. |
| 9 | Chana (2020) | England | Qualitative |  | 1 |  | 1 |  |  | Blending in (1), routine modification (2),  containment (1) | | | 4 | Response to and experience of hate  crimes victimisation | No | .. |

**Appendix 2** (continued)

Descriptives of studies on personal adaptations

| **Author and publication date** | **Country (data)** | **Method** | **Religion (preventing victimisation against:)** | | | | | | **Main adaptation / intervention type**** | **Number of measures frequency** | **Specification of main outcome** | **RCT** | **Result if RCT** |
| --- | --- | --- | --- | --- | --- | --- | --- | --- | --- | --- | --- | --- | --- |
|  |  |  | *Christ.* | *Islam* | *Judais* | *Asian* | *Other* | *Nonrel.* |  |  |  |  |  |

| 10 | Cieslik &  Phillips (2021) | USA | Qualitative | 1 | Blending in (2), routine modification (1) | 3 | Perceived safety from  hate crime victimisation | No | .. |
| --- | --- | --- | --- | --- | --- | --- | --- | --- | --- |
| 11 | Flax (2021) | England | Qualitative | 1 | Religion as source of resilience (2), containment (1) | 3 | Response to and experience of hate crimes  victimisation | No | .. |
| 12 | Gampiot (2017) | Binational | Qualitative | 1 | Blending in (3), religion as source of  resilience (1), routine modification (1) | 5 | Perceived safety from  hate crime victimisation | No | .. |
| 13 | Hargreaves  (2016) | Binational | Qualitative | 1 | Religion as source of resilience (1) | 1 | Perceived safety from  hate crime victimisation | No | .. |
| 14 | Ljamai (2020) | Netherlands | Qualitative | 1 | Counterattack (1) | 1 | Anxiety by radicalised  youth | No | .. |
| 15 | Mercier- Dalphond &  Helly (2021) | Canada | Qualitative | 1 | Routine modification (2), religion as source of resilience (1) | 3 | Perceived safety from hate crime victimisation | No | .. |
| 16 | Morlock (2021) | USA | Qualitative | 1 | Blending in (1), religion as source of  resilience (1) | 2 | Perceived safety from  hate crime victimisation | No | .. |
| 17 | O'Brien (2011) | USA | Qualitative | 1 | Blending in (3), containment (1), religion as source of resilience (1) | 5 | Response to and experience of hate crimes victimisation, Perceived  safety from hate crime victimisation | No | .. |
| 18 | Perry (2015) | Canada | Qualitative | 1 | Religion as source of resilience (2) | 2 | Perceived safety from  hate crime victimisation | No | .. |

**Appendix 2** (continued)

Descriptives of studies on personal adaptations

|  | **Author and publication date** | **Country (data)** | | **Method** | **Religion (preventing victimisation against:)** | | | | | | **Main adaptation / intervention type**** | **Number of measures frequency** | | **Specification of main outcome** | **RCT** | **Result if RCT** | |
| --- | --- | --- | --- | --- | --- | --- | --- | --- | --- | --- | --- | --- | --- | --- | --- | --- | --- |
|  |  |  |  |  | *Christ.* | *Islam* | *Judais* | *Asian* | *Other* | *Nonrel.* |  |  |  |  |  |  |  |
| 19 | Saleem et al. (2022) | USA | Qualitative | | 1 | |  |  |  | Blending in (1), routine  modification (1), religion as source of resilience (3) | | | 5 | Response to and experience of victimisation | | No | .. |
| 20 | Wigerfelt &  Wigerfelt (2016) | Sweden | Qualitative | |  | 1 |  |  |  | Blending in (2), routine  modification (2) | |  | 4 | Response to and experience  of hate crimes victimisation | | No | .. |
| 21 | Yeste et al.  (2020) |  |  | |  |  |  |  |  | Blending in (1), routine  modification (1) | |  |  | Perceived safety from hate  crime victimisation | |  |  |
|  |  | Spain | Qualitative | | 1 |  |  |  |  |  |  |  | 2 |  |  | No | .. |
| 22 | Zempi (2020) | UK | Qualitative | | 1 |  |  |  |  | Blending in (1), routine modification (1), containment (1),  counterattack (1) | | | 4 | Response to and experience of hate crimes victimisation | | No | .. |
| N | 22 |  |  | *1* | *15* | *5* |  | *1* | *1* | *1* |  |  | 64 |  | | 0 |  |
| % | 100 |  |  | 4% | 57% | 19% |  | 12% | 4% | 4% |  |  |  |  | |  |  |
| * Two different Asian religions | | | | | |  |  |  |  |  |  |  |  |  | |  |  |
| ** Refers to the number of different measures inside the main category | | | | | |  |  |  |  |  |  |  |  |  | |  |  |

# Appendix 3

Descriptives of studies on collective/community programs

|  | **Author and publication date** | **Country (data)** | **Method** | **Religion (preventing victimisation against:)** | | | | | | **Main adaptation / intervention type** | **Number of measures frequency** | **Specification of main outcome** | **RCT** | **Result if RCT** |
| --- | --- | --- | --- | --- | --- | --- | --- | --- | --- | --- | --- | --- | --- | --- |
|  |  |  |  | *Christ.* | *Islam* | *Judais* | *Asian* | *Other* | *Nonrel* |  |  |  |  |  |
|  |  |  |  |  |  |  |  |  |  | Mobilising community  resilience (4), Increasing positive inter-group contact (1) | 5 | Response to and experience of hate  crimes victimisation (in a collective way) | No | **..** |
| 1 | Agrawal et al. (2019) | USA | Qualitative |  | 1 |  |  |  |  |  |  |  |  |  |
| 2 | Alrababa'h et al. (2021) | England | Quantitative  (survey & experiment) |  | 1 |  |  |  |  | Stereotype reduction (1) | 1 | Prejudice reduction | Yes | Effective |
| 3 | Bacchus (2019) | USA | Qualitative |  | 1 |  |  |  |  | Mobilising community resilience (3) | 3 | Response to and experience of hate crimes victimisation (in a  collective way) | No | .. |
| 4 | Ben-Moshe & Halafoff  (2014) | Australia | Qualitative |  |  | 1 |  |  |  | School-based prevention (2) | 2 | Response to and experience of hate crimes victimisation (in a  collective way) | No | .. |
| 5 | Berger et al. (2016) | Israel | Quantitative (survey & experiment) | |  | 1 |  |  |  | School-based prevention (1) | 1 | Prejudice reduction | Yes | Effective |
| 6 | Bruneau et al. (2018) | USA | Quantitative (Intervention  tournament) |  | 1 |  |  |  |  | Stereotype reduction (1) | 1 | Prejudice reduction | Yes | Effective |
| 7 | Dhali et al.  (2022) | Canada | Mixed | 1 | 1 | 1 | 2 | 1 | 1 | School-based prevention (1) | 1 | Radicalised thinking reduction,  prejudice reduction | No | .. |
| 8 | Dhattiwala  (2016) | India | Qualitative (multi) |  | 1 |  | 1 |  |  | Place-based prevention (2) | 2 | Hate crime reduction (in a violent  conflict) | No | .. |
| 9 | Galily, et al. (2013) | Israel | Quantitative  (survey & experiment) | 1 | 1 | 1 | |  |  | Increasing positive inter-group contact (2) | 2 | Prejudice reduction | Yes | Effective |

# Appendix 3 (continued)

Descriptives of studies on collective/community programs

| **Author and publication date** | **Country (data)** | **Method** | **Religion (preventing victimisation against:)** | | | | | | **Main adaptation / intervention type** | **Number of measures frequency** | **Specification of main outcome** | **RCT** | **Result if RCT** |
| --- | --- | --- | --- | --- | --- | --- | --- | --- | --- | --- | --- | --- | --- |
|  |  |  | *Christ.* | *Islam* | *Judais* | *Asian* | *Other* | *Nonrel.* |  |  |  |  |  |

| 10 | Gallardo et al.  (2021) | Binational | Quantitative (survey  & experiment) | | 1 |  | Stereotype reduction (1) | 1 | Prejudice reduction | Yes | Effective |
| --- | --- | --- | --- | --- | --- | --- | --- | --- | --- | --- | --- |
| 11 | McCauley (2014) | Côte d'Ivoire | Quantitative (survey &  experiment) | 1 | 1 |  | Stereotype reduction (1) | 1 | Prejudice reduction | No | .. |
| 12 | Mercier-  Dalphond & Helly (2021) | Canada | Qualitative |  | 1 |  | Mobilising community resilience (2) | 2 | Response to and experience of hate  crimes victimisation (in a collective way) | No | .. |
| 13 | Moore-Berg et al. (2022) | USA | Quantitative (Intervention  tournament) |  | 1 |  | Stereotype reduction (1) | 1 | Prejudice reduction | Yes | Effective |
| 14 | Moritz et al. (2017) | Germany | Quantitative (survey &  experiment) | 1 | 1 | 1 | Mobilising community resilience (1) | 1 | Prejudice reduction | Yes | Effective |
| 15 | Murrar & Brauer (2018) | USA | Quantitative (survey & experiment) | | 1 |  | Stereotype reduction (2), mobilising community  resilience (1) | 3 | Prejudice reduction | Yes | Effective |
| 16 | O'Brien (2011) | USA | Qualitative |  | 1 |  | Mobilising community resilience (1) | 1 | Response to and experience of hate crimes victimisation (in a  collective way) | No | .. |
| 17 | Orjuela (2020) | Binational | Qualitative |  | 1 | 1 | Increasing positive inter-group contact (1), Mobilising  community resilience (1) | 2 | Religious peacebuilding | No | .. |
| 18 | Ozalp et al. (2020) | UK | Quantitative (supervised machine  learning) | |  | 1 | Mobilising community resilience (1) | 1 | Countering online antisemitism | No | .. |

# Appendix 3 (continued)

Descriptives of studies on collective/community programs

| **Author and publication date** | **Country (data)** | **Method** | **Religion (preventing victimisation against:)** | | | | | | **Main adaptation / intervention type** | **Number of measures frequency** | **Specification of main outcome** | **RCT** | **Result if RCT** |
| --- | --- | --- | --- | --- | --- | --- | --- | --- | --- | --- | --- | --- | --- |
|  |  |  | *Christ.* | *Islam* | *Judais* | *Asian* | *Other* | *Nonrel.* |  |  |  |  |  |

| 19 | Pavetich & Stathi (2021) | Binational | Quantitative  (survey & experiment) | 1 |  | 1 | 1 | 1 | Stereotype reduction (1) | 1 | Prejudice reduction | Yes | Effective |
| --- | --- | --- | --- | --- | --- | --- | --- | --- | --- | --- | --- | --- | --- |
| 20 | Perry (2015) | Canada | Qualitative |  | 1 |  |  |  | Stereotype reduction (1) | 1 | Response to and experience of hate crimes victimisation (in a  collective way) | No | .. |
| 21 | Saleem et al. (2022) | USA | Qualitative |  | 1 |  |  |  | Mobilising community resilience (2) | 2 | Response to and experience of hate  crimes victimisation (in a collective way) | No | .. |
| 22 | Savage et al. (2014) | Kenya | Quantitative  (survey & experiment) | 1 | 1 |  |  | 1 | School-based prevention (1) ,  mobilising community resilience (1) | 2 | Radicalised thinking reduction | Yes | Effective |
| 23 | Savani et al.  (2020) | USA | Quantitative (survey  & experiment) | | 1 |  |  |  | School-based prevention (1) | 1 | Prejudice reduction | Yes | Effective |
| 24 | Scheitle  (2018) | USA | Quantitative  (survey) | 1 | 1 | 1 | 2 |  | Place-based prevention (2) | 2 | Perceived safety from hate crime  victimisation | No | .. |
| 25 | Scheitle &  Ulmer (2018) | USA | Qualitative  (interviews) |  |  |  | 3 | 1 | Place-based prevention (5) | 5 | Perceived safety from hate crime  victimisation | No | .. |
| 26 | Shankar &  Gerstein (2007) | India | Qualitative (interviews) |  | 1 |  | 1 |  | Increasing positive inter-group contact (1) | 1 | Perceived hate crime | No | .. |
| 27 | Siem et al.  (2021) | Germany | Quantitative (survey  & experiment) | | 1 |  |  |  | Stereotype reduction (1) | 1 | Prejudice reduction | Yes | Effective |
| 28 | Yeste et al. (2020) | Spain | Qualitative |  | 1 |  |  |  | Mobilising community resilience (1) | 1 | Response to and experience of hate  crimes victimisation (in a collective way) | No | .. |

# Appendix 3 (continued)

Descriptives of studies on collective/community programs

| **Author and publication date** | **Country (data)** | **Method** | **Religion (preventing victimisation against:)** | | | | | | **Main adaptation / intervention type** | **Number of measures frequency** | **Specification of main outcome** | **RCT** | **Result if RCT** |
| --- | --- | --- | --- | --- | --- | --- | --- | --- | --- | --- | --- | --- | --- |
|  |  |  | *Christ.* | *Islam* | *Judais* | *Asian* | *Other* | *Nonrel.* |  |  |  |  |  |

| 29 | Zempi (2020) | UK | Qualitative | 1 |  |  |  | Mobilising community resilience (1) | 1 | Response to and experience of hate crimes victimisation (in a  collective way) | No | .. |
| --- | --- | --- | --- | --- | --- | --- | --- | --- | --- | --- | --- | --- |
|  |  |  | *7* | *24* | *8* | *11* | *2* | *3* | |  |  |  |
| N | 29 |  |  |  |  |  |  |  | 49 |  | 12 |  |
|  |  |  | 13% | 43% | 15% | 20% | 4% | 5% | |  |  |  |
| % | 100 | | | | | | |  | |  | 41% |  |
| * Two different Asian religions | | | | | | | |  | |  |  |  |
| ** Refers to the number of different measures inside the main category | | | | | | | |  | |  |  |  |

# Appendix 4

Descriptives of studies on institutional-governmental policies

|  | **Author and publication date** | **Country (data)** | **Method** | **Religion (preventing victimisation against:)** | | | |  |  | **Main adaptation**  **/ intervention type** | **Number of measures**  **frequency** | **Specification of main outcome** | **RCT** | **Result if RCT** |
| --- | --- | --- | --- | --- | --- | --- | --- | --- | --- | --- | --- | --- | --- | --- |
|  |  |  |  | *Christ.* | *Islam* | *Judaism* | *Asian* | *Other* | *Nonrel.* |  |  |  |  |  |
| 1 | Chana (2020) | England | Qualitative |  | 1 |  | 1 |  |  | Police- community  relations (2) | 2 | Perceived safety from hate crime victimisation | No | .. |
| 2 | Hardy et al. (2020) | UK | Qualitative |  |  |  |  |  |  | Police training  (1) | 1 | Improvement of the police  response to hate crime | No | .. |
| 3 | Jurczak (2019) | Poland | Mixed | 1 | 1 | 1 | 2 | 1 | 1 | Police training  (1) | 1 | Improvement of the police  response to hate crime | No | .. |
| N | 3 |  |  | *1* | *2* | *1* | *3* | *0* | *1* |  | 4 |  | 0 |  |
|  |  |  |  | 13% | 25% | 13% | 36% | 0% | 13% |  |  |  |  |  |
| % | 100 | | | | |  |  |  |  |  |  |  | 0% |  |
| * Two different Asian religions | | | | | |  |  |  |  |  |  |  |  |  |
| ** Refers to the number of different measures inside the main category | | | | | |  |  |  |  |  |  |  |  |  |
